# Morphological Characterisation of Unstained and Intact Tissue Micro-architecture by X-ray Computed Micro- and Nano-Tomography

**DOI:** 10.1038/srep10074

**Published:** 2015-05-15

**Authors:** Lucy A. Walton, Robert S. Bradley, Philip J. Withers, Victoria L. Newton, Rachel E. B. Watson, Clare Austin, Michael J. Sherratt

**Affiliations:** 1 Institute of Cardiovascular Sciences; 2 School of Materials; 3 Institute of Inflammation and Repair, University of Manchester, Manchester, United Kingdom; 4 Faculty of Health and Social Care, Edge Hill University, Ormskirk, United Kingdom

## Abstract

Characterisation and quantification of tissue structures is limited by sectioning-induced artefacts and by the difficulties of visualising and segmenting 3D volumes. Here we demonstrate that, even in the absence of X-ray contrast agents, X-ray computed microtomography (microCT) and nanotomography (nanoCT) can circumvent these problems by rapidly resolving compositionally discrete 3D tissue regions (such as the collagen-rich adventitia and elastin-rich lamellae in intact rat arteries) which in turn can be segmented due to their different X-ray opacities and morphologies. We then establish, using X-ray tomograms of both unpressurised and pressurised arteries that intra-luminal pressure not only increases lumen cross-sectional area and straightens medial elastic lamellae but also induces profound remodelling of the adventitial layer. Finally we apply microCT to another human organ (skin) to visualise the cell-rich epidermis and extracellular matrix-rich dermis and to show that conventional histological and immunohistochemical staining protocols are compatible with prior X-ray exposure. As a consequence we suggest that microCT could be combined with optical microscopy to characterise the 3D structure and composition of archival paraffin embedded biological materials and of mechanically stressed dynamic tissues such as the heart, lungs and tendons.

Characterising tissue sub-structure by conventional histological sectioning (microtomy) frequently induces tears, fractures, compressions and folds^1^ and, in the specific case of large arteries, often causes detachment of the adventitial layer. Crucially, in order to accurately characterise the structure of tissues such as elastic arteries, it will also be necessary to image sufficiently large regions of the vessel in three-dimensions (3D)^2^,^3^. Although recent advances in serial block face scanning electron microscopy and confocal laser microscopy have been employed to visualise the 3D structure of the aortic medial layer^3^ and adventitial collagen^4^, these techniques can only be applied to relatively small tissue volumes. However, X-ray computed microtomography (microCT) which is conventionally used for determining the structure of hard calcified tissues such as bone, can also be used to visualise the 3D morphology of intact non-calcified organs and organisms in the presence of X-ray contrast agents^5^,^6^. In this study we aimed to determine if sub-μm tissue-structures (in large arteries and skin) could be visualised in the absence of exogenous X-ray contrast agents.

Large conduit arteries protect smaller vessels from damaging variations in blood pressure^7^. This mechanical dampening function is mediated primarily by cross-linked elastin in the medial elastic lamellae and by collagen fibrils in the outer adventitial layer^8^. Aberrations in this wall structure, as a consequence of heritable connective tissue disorders, ageing and diseases such as diabetes, are associated with life-threatening cardiovascular events including heart failure and aortic aneurysm^9^,^10^,^11^. In order therefore to characterise both the pathological progression of these disorders and the efficacy of potential treatments it is important to visualise arterial wall structure. In practice this is commonly achieved by two dimensional sectioning of unpressurised vessels and subsequent characterisation of the medial layer only. In pioneering work published in 1964, Wolinsky and Glagov documented acute reductions in elastic lamellae “waviness” and medial layer thickness with increasing intra-luminal pressure^12^. In their model system, the New Zealand White Rabbit, the majority of these acute tissue remodelling events took place at sub-physiological pressures^13^ of less than 80 mm Hg. Despite this clear evidence that the structure of the pressurised vessel differs profoundly from the structure of the unpressurised vessel (a state which only occurs after death) and that the outer adventitial layer also plays a vital role in limiting vessel distension^14^ and preventing aneurysms^15^, with few exceptions^3^,^4^,^16^,^17^ arterial morphology is still routinely characterised in 2D from sectioned, unpressurised, tissues.

Here, we characterise the structure of intact Wistar rat large arteries (preserved in either an unpressurised or pressurised state) using microCT. We demonstrate that this technique can differentiate between sub-tissue level layers and that intra-luminal pressure results in profound remodelling of both the medial and adventitial layers. In future studies, it will be important to progress from the static application of intra-luminal pressure (in which water flow may influence tissue architecture and volume over long time scales) to dynamic x-ray imaging of native arteries subjected to physiological cyclic loading regimes. We also demonstrate that X-ray micro-tomography is applicable to other organs such as human skin and that sufficient contrast can be achieved without the use of X-ray contrast agents (which can interfere with subsequent histological and immunohistochemical analyses). As a consequence the technique will be applicable to most archival paraffin embedded biological material.

## Results and discussion

### *Microtomography Preserves Arterial Structure and Resolves Compositionally Distinct Tissue Regions and Components*

**Figure 1:**
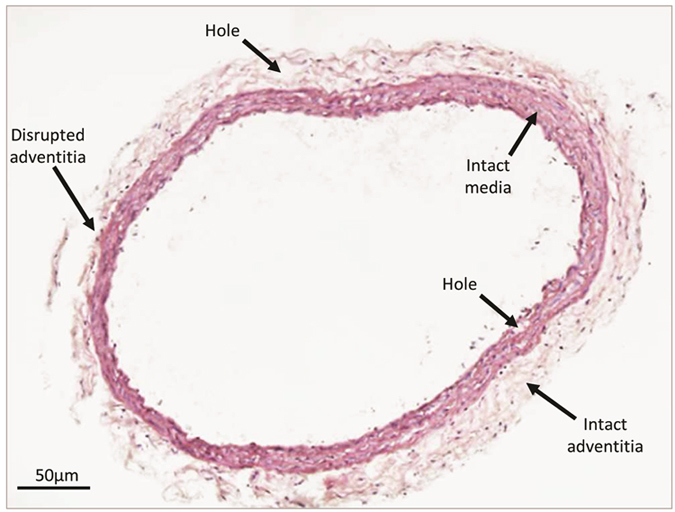
Sectioning-induced artefacts in paraffin embedded vascular and cutaneous tissues. Light microscopy image of H&E stained rat CCA.

**Figure 2:**
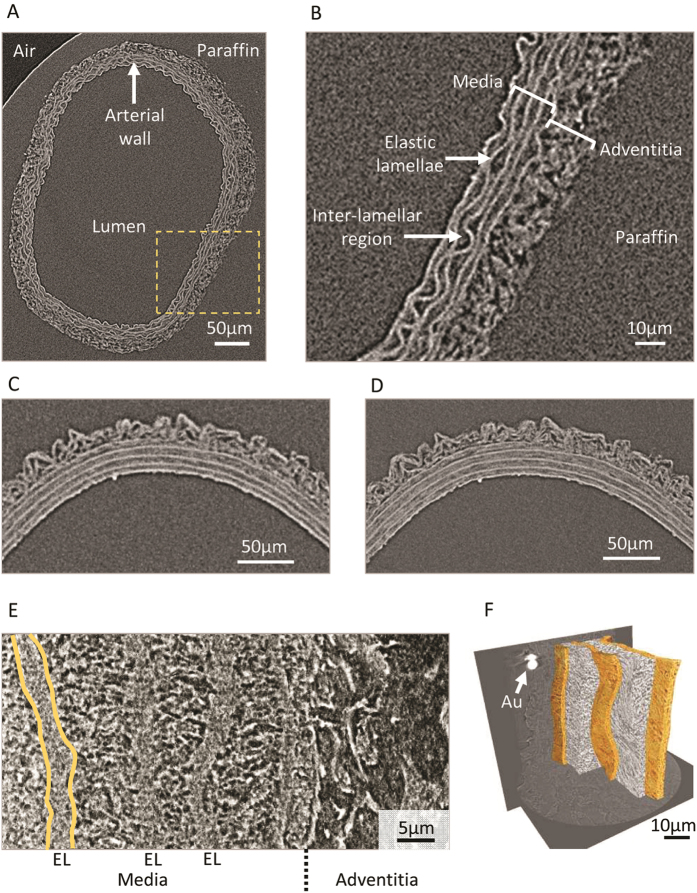
Sub-micron X-ray tomography resolves discrete tissue regions and components in paraffin embedded arteries. (**A**) Virtual slice extracted from an X-ray tomogram of an intact rat CCA (yellow box indicates magnified region in panel B). Even in the absence of exogenous X-ray contrast agents, the X-ray density of native soft tissues is higher than that of both paraffin and air. (**B**) Major arterial sub-structures are readily identifiable in the vessel wall. (**C** and **D**) The resolution achievable with a 4x objective lens (**C**: voxel size 0.75 μm, 2D resolution 1.2 μm) can be improved using a 20x objective (**D**: voxel size 0.50 μm, 2D resolution 0.7 μm). (**E**) Virtual slice extracted from an X-ray tomogram at nanoscale resolution (150 nm) of an intact rat aorta. The tomogram was taken with Zernike phase contrast which gives contrast to edge features and resolves fibrous structures within the inter-lamellar regions. (**F**) Elastic lamellae and inter-lamellar regions can be readily segmented and gold particles (Au) can be used to locate the same tissue region on differing instruments.

Histological analysis of tissue structure is most commonly performed on thin (<10 μm) sections cut from formalin fixed and paraffin embedded samples^18^. Although the formalin-induced amine cross-links and paraffin wax act in concert to chemically strengthen and physically support the tissue^19^, the sectioning process invariably disrupts the structure of organs such as large arteries. As a consequence vascular tissue sections are commonly characterised by folds, holes (primarily, although not exclusively, located in the adventitia) and by separation of the collagen-rich adventitia from the medial external elastic lamina (Fig. 1). By avoiding mechanical sectioning, X-ray microCT is capable of preserving the 3D structure of whole organisms and organs^5^,^6^. Here we show that X-ray microCT can also visualise structurally intact and compositionally distinct regions and macro-molecular assemblies in blood vessels at an approximate spatial resolution of 1.2 μm (Fig. 2A,B) when using a 4x objective lens or 0.7 μm when using a higher power 20x lens (Fig. 2C,D). Due to the highly organised architecture of large arteries we can show that common tissue components, the fibrillar collagen-rich adventitia, elastin-rich elastic lamellae and cell-rich inter-lamellar regions, differ in their relative X-ray opacities and can therefore be visualised by this technique. Although in isolation, individual virtual X-ray tomogram slices (such as those depicted in Fig. 2A–D) contain a wealth of structural information, each slice represents only a fraction of the 3D X-ray density data which is encoded in the complete reconstruction. By viewing the 3D data as sequential 2D projections in an animated Video (Video S1) new structural information, such as the topography of the luminal surface, becomes clear. Crucially, although perturbations in the roughness of this surface are thought to play an important role in promoting localised vascular damage (and hence cardiovascular diseases such as atherosclerosis^20^,^21^), conventional sectioning methods are unable to visualise the 2D micro-topography of the luminal intima/blood interface.

Until recently, x-ray tomography has been limited in its ability to reveal features within non-calcified, and hence weakly x-ray absorbing, tissues. Whilst x-ray absorption and therefore contrast can be enhanced using high Z elements^22^ difficulties remain with ensuring that these agents: i) penetrate larger tissues, ii) differentiate between tissue components and iii) are compatible with subsequent histological techniques^23^. Alternatively, x-ray phase contrast approaches can enhance contrast in non-calcified tissues including cartilage^24^, tendon^25^, plaque containing blood vessels^26^, coronary artery^27^, ligaments and the intervertebral disc^28^. However, such approaches may require complex imaging set-ups^29^ and post-processing^30^, and image acquisition times can be long for laboratory instruments. In this paper, we demonstrate that excellent differential contrast can be achieved in non-calcified tissues with a laboratory microCT system using absorption contrast in conjunction with phase contrast enhancement. We attribute this improved contrast to both our specimen preparation techniques and the instrumentation. Tissue samples were prepared using a standard histological procedure which involves chemical fixation, alcohol dehydration and infiltration with paraffin wax. Differential dehydration of tissue components (such as low-density, hydrated cells and densely packed hydrophobic elastic lamellae in large arteries) in combination with localised paraffin infiltration may increase internal contrast. Instrumental improvements include the use of thin scintillators which absorb fewer higher energy x-rays than conventional flat panel detectors^31^. These high energy x-rays do not contribute significantly to contrast of low Z materials but rather to the background noise. Furthermore, flat panel detectors may contain an Al filter which would further reduce the relative contribution from low energy x-rays to image formation, making them suitable for x-ray energies greater than ~40 keV^32^. Finally, in-line phase contrast enhances contrast at the multiple boundaries (such as lumen-wall and elastic lamellae-interlamellar regions) which are found in tissues such as arteries thereby providing additional edge-enhancement^30^.

### *The Complex Architecture of Medial Layer Interlamellar Spaces is Evident in Nanotomography Reconstructions*

Whilst the X-ray micro-tomograms depicted in panels A-D of Fig. 2 are highly informative, most organs and tissue are organised at multiple length scales. In order therefore to resolve, for example, fine elastic fibres, collagen fibril bundles and vascular smooth muscle cell structure within the arterial medial lamellar unit^3^,^33^ it is necessary to improve on the 0.5 μm resolution which can be achieved by using the Carl Zeiss XRM Xradia Versa microCT. Sub-100 nm resolutions can be achieved with both synchrotron and laboratory X-ray sources^34^ and by using concepts pioneered in optical microscopy (such as Fresnel zone plates) X-rays can be focused with phase contrast achieved by propagation phase contrast or Zernike phase contrast phase rings^35^. These techniques have previously been used to image unstained bacteria, insects and mineralised tissues^36^,^37^. Here we demonstrate using Zernike phase contrast on a Carl Zeiss XRM Xradia Ultra-810 (with a resolution of 150 nm) that, compared with the amorphous appearance which characterises medial interlamellar regions in micro-tomograms, these same areas appear structurally heterogeneous by nanotomography (nanoCT) (Fig. 2B,D). This dense fibrous network may be composed of discrete, small-diameter, elastic fibres and collagen fibril bundles^3^,^33^. If necessary, tissue structure could be studied across multiple length scales by labelling a region of interest with gold spheres (as depicted by the area of bright contrast in Fig. 2F) hence enabling cross-correlative tomographic imaging of the same sample region by microCT and nanoCT. In order to fully exploit the wealth of 3D structural data which is present in both this nanoCT reconstruction and microCT data sets, we have developed semi-automatic image analysis approaches to segment structurally and compositionally distinct, yet low-contrast, tissue regions.

### *Tissue-Specific Segmentation Strategies are Required to Quantitatively Characterise 3D tissue Structure*

**Figure 3:**
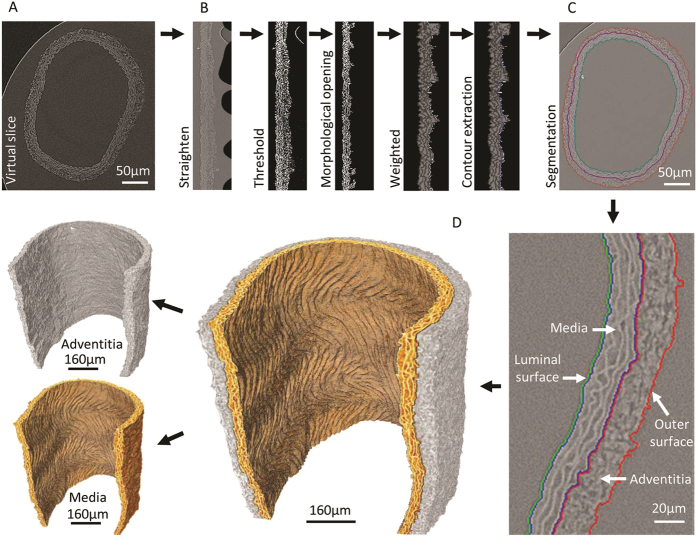
Semi-automatic segmentation of medial and adventitial layers and elastic lamellae in large arteries. (**A**) Virtual trans-axial slice through an X-ray tomogram of a rat CCA obtained by micro CT. (**B**) Segmentation of the medial and adventitial layers was performed based on differences in grey level texture using the following steps: i) unwrapping to straighten out the wall, ii) morphological opening and island removal so that only features which are aligned mostly along the vertical direction are retained (most features within the adventitia are removed) and iii) extraction of the contour between the medial and adventitial layers. This is based on an optimization method to find the geodesic path through a weight image. (**C**) The contour is re-wrapped onto the original slice. (**D**) Rendering showing the output of the segmentation process which enables the medial and adventitial layers to be virtually dissected.

**Figure 4:**
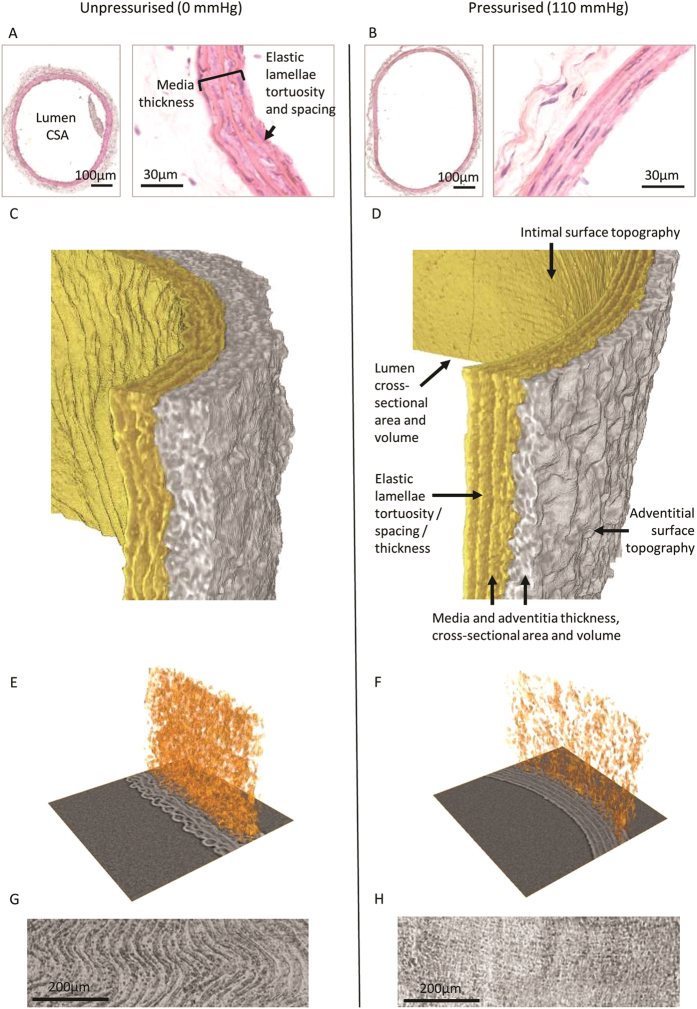
Intra-luminal pressure-induced remodelling of rat common carotid arteries. (**A** and **B**) Sections of vessels preserved unpressurised (**A**) and pressurised (**B**) states. (**C** and **D**) X-ray tomography reconstructions of unpressurised (**C**) and pressurised (**D**) vessels. The medial and adventitial layers are segmented in yellow and in grey respectively. (**E** and **F**) Segmented pores (with an X-ray density lower than paraffin: depicted as orange 3D volume projections) from unpressurised (**E**) and pressurised arteries (**F**) mapped onto a 2D circumferential slice. (**G** and **H**) 3D projections of unwrapped luminal surfaces extracted from reconstructions of unpressurised (**G**) and pressurised (**H**) vessels. The unpressurised surface is characterised by a rippled appearance which is oriented along the axis of the vessel.

**Figure 5:**
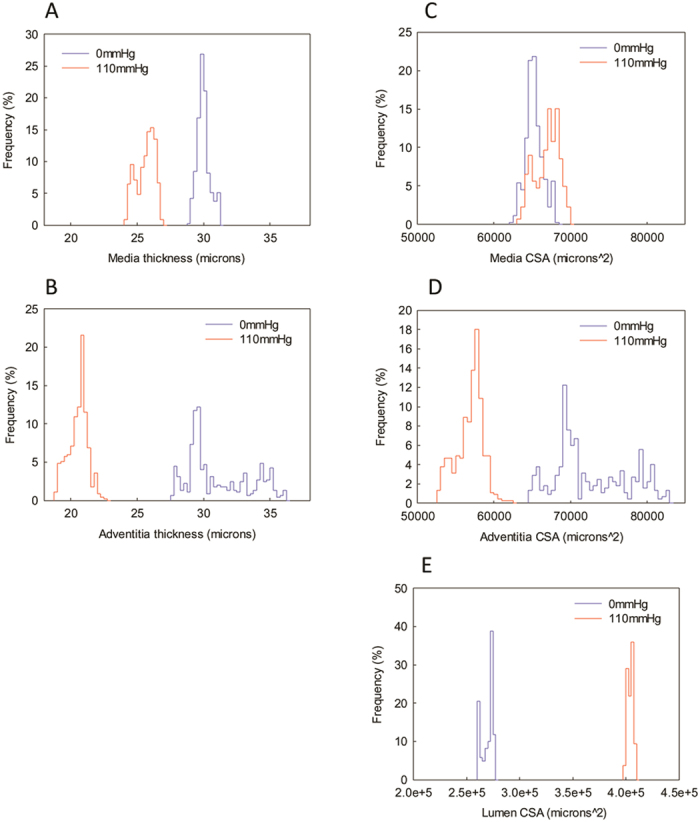
Morphological characterisation of medial and adventitial layer dimensions in unpressurised and pressurised arteries. Frequency distribution histograms of mean medial layer thickness (**A**) and adventitial layer thickness (**B**). The cross-sectional area (CSA) of the medial (**C**) and adventitial (**D**) layers and the lumen (**E**) can be rapidly calculated from segmented X-ray tomograms. Thicknesses and areas are derived from a large tomographic volume (450 axial slices, axial length 338 μm).

Large arteries are highly structured tissues in which the major ECM components, elastin and fibrillar collagens, are both X-ray opaque (compared with the paraffin support) and largely confined to the medial elastic lamellae and adventitial layers respectively (Fig. 1,2). Additionally, the elastic lamellae confer an ordered structure on the medial layer which contrasts with the less-ordered adventitial layer. We have developed a sequential image processing protocol which exploits differences in X-ray opacity in order to segment the vessel wall from the supporting paraffin^38^,^39^ and then uses the morphological characteristics^40^ of the elastic lamellae to segment the media from the adventitia (Fig. 3). By automatically segmenting 3D X-ray tomography volumes, we have been able to: i) avoid potential sampling errors and gain insight into variations in morphology by rapidly characterising these morphological parameters at all points around the vessel cross-section in each of the 450 virtual slices (4x objective), ii) characterise the morphology of the intact adventitial layer and iii) assess the effects of intra-luminal pressure on the topography of clinically important interfaces such as that formed between the luminal surface and the blood stream^41^ (Figs. 4 and 5). A similar approach can be used to segment distinct 3D structures within the medial layer of nanoCT reconstructions (Fig. 2E,F). Having established methods for visualising and measuring the 3D volume and topography of discrete tissue regions and surfaces we can use these approaches to quantify the effects of intra-luminal pressure on the structure of large arteries.

### *MicroCT Preserves Arterial Structure and Allows for the Measurement of Novel Morphological Characteristics*

In 1964 Wolinsky and Glagov reported on the effects of intra-luminal pressure on the 2D architecture of the aortic media^12^. Here we show that, compared with the unpressurised vessel, the pressurised (at 110 mm Hg) rat common carotid artery (CCA) also exhibits significant structural remodelling (Fig. 4). From the segmented volume, it is apparent that both medial (0 mm Hg: 29.99 ± 0.02 μm, 110 mm Hg: 25.62 ± 0.69 μm, p < 0.0001) and adventitial (0 mm Hg: 31.12 ± 0.11 μm, 110 mm Hg: 20.55 ± 0.03 μm, p < 0.0001) layer thickness are significantly lower in the pressurised vessel (Fig. 5A,B). By segmenting the 3D X-ray tomogram data we can additionally measure the cross-sectional area (CSA) of the lumen and medial and adventitial layers in each of the 450 slices in the tomogram (Fig. 5C–E). Wolinksy and Glagov^12^ reported a pressure-induced non-linear increase in vessel radius and a decrease in medial layer thickness, here we show that intra-luminal pressure is associated with a greater lumen CSA (0 mm Hg: 270,030 ± 256 μm^2^, 110 mm Hg: 404,043 ± 12^2^ μm^2^, p < 0.0001). By preserving both major structural layers of the artery we additionally show that the adventitial layer is less uniform in morphology than the medial layer and that the numerous pores (identified by their low X-ray density compared with paraffin wax) which characterise the unpressurised adventitia are considerably reduced in volume and abundance in the pressurised vessel (Fig. 4E,F). As a consequence of this remodelling, the cross-sectional area (CSA) of the adventitia is significantly reduced in the pressurised vessel (medial CSA: 0 mm Hg: 65,258 ± 53 μm^2^, 110 mm Hg: 66,922 ± 76 μm^2^, p < 0.0001; adventitial CSA: 0 mm Hg: 72,658 ± 228 μm^2^, 110 mm Hg: 56,844 ± 82 μm^2^, p < 0.0001). Crucially however, it remains to be determined if comparable adventitial remodelling would be evident either between physiological intra-luminal pressures and/or in dynamic, rather than static, loading conditions. In order to understand the function of healthy and diseased arteries and to design appropriate tissue engineered replacements it will be necessary to model the mechanical behaviour of arterial tissues^42^. MicroCT has the potential to contribute to these models by identifying localised remodelling events in mechanically deformed cardiovascular tissues and in other organs such as aged and hence fragile skin^43^.

### *MicroCT can Visualise Tissue Sub-Structures in Other ECM-Rich Organs*

**Figure 6:**
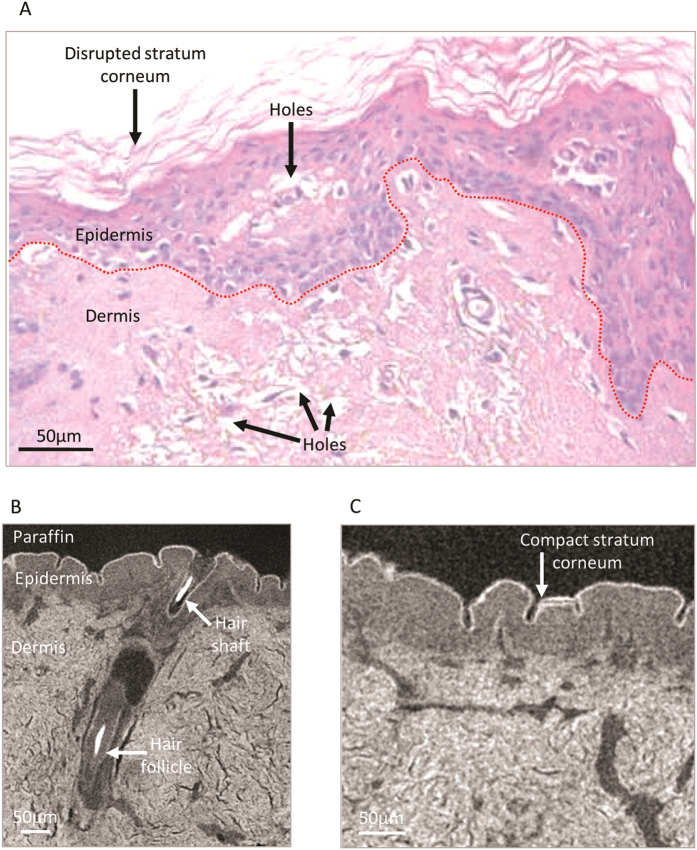
Sectioning induced artefacts and sub-micron X-ray tomography of human skin. (**A**) H&E stained human skin biopsy. The dermal-epidermal junction is denoted by a red dotted line. (**B** and **C**) Virtual slices extracted from an X-ray tomogram of a human skin biopsy. The dermal and epidermal layers and large structures such as hair follicles are clearly visible and outer *stratum corneum* remains densely packed and closely adhered to the underlying tissue.

Skin is an organ which is composed of two main tissue layers: an outer cell-rich and avascular epidermis and a supporting ECM-rich dermis which also contains an extensive microvasculature and discrete structures such as hair follicles. As with large arteries, skin is prone to sectioning induced artefacts (Fig. 6A). Whilst in skin, the epidermis rarely separates from the dermis, both tissue regions are subject to sectioning-induced tears and the separation and delamination of the outer *stratum corneum*. In contrast, microCT reconstructions clearly show a closely adhered *stratum corneum* (Fig. 6B,C). This densely packed barrier layer (which is composed primarily of extracellular lipids and disulphide-bonded intracellular keratins), the cellular epidermis, the ECM-rich dermis, hair follicles and potentially a branched micro-vasculature can all be resolved by virtue of their relative X-ray density and architecture (Fig. 6B,C). The 3D architecture of each reconstruction may also be examined by virtual sectioning of the volume at arbitrary angles to visualise, for example, the structure of skin perpendicular (Video S2) and parallel with the surface (Video S3). In this latter video the rhomboidal organisation of the *stratum corneum*, which is not evident in histological sections, can be clearly seen as X-ray dense circles formed by cross-linked keratin-rich hair shafts. To our knowledge this is the first demonstration that microCT can visualise such skin sub-structures. Previously, microCT has been employed to either visualise skin in relation to other organs (whole limb microCT in mice)^44^ or to localise foreign particles such as gunshot residues^45^.

### *Histological and Immunohistochemical Procedures are Compatible with microCT*

**Figure 7:**
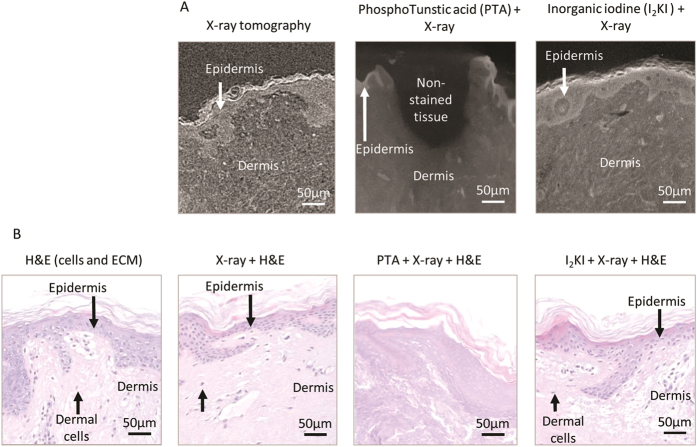
The use of contrast agents in the skin with X-ray tomography. Using the Carl Zeiss Versa-510 system unstained skin may be visualised. However, contrast is low and the differentiation of structures within the skin is difficult. The use of PTA or I_2_KI contrasting agents improves the differentiation obtained between structures in the skin. PTA provides the best contrast when looking at structures within the skin, however, due to its molecular size, its rate of staining is slow and full penetration of the tissue is not achieved, even after many hours (**A**). Further to this, if tissue that has been imaged using X-ray tomography is then sectioned and stained with H&E to visualise skin structure, the expected staining pattern is disrupted by the presence of PTA. However, X-ray exposure and the use of I_2_KI contrasting agent does not prevent successful H&E staining of skin sections (**B**).

It is clear from the data presented in Fig. 2, 3, 4, 5, 6 that even in the absence of exogenous X-ray contrast agents X-ray tomography can resolve sub-structures in chemically fixed, paraffin embedded samples. By combining X-ray tomography imaging with subsequent histological^46^ and immunohistochemical analyses it should be possible to confirm the identity of discrete structures in X-ray tomograms. Exposure to synchrotron- derived X-rays has been shown to induce thermal and/or oxidative damage to structural protein such as collagen^47^. In this study we examined the effects of X-ray exposure alone (in our microCT system) and X-ray exposure in combination with X-ray contrast agents on the binding of common histological stains to skin sections. Haematoxylin and eosin (H&E) staining is widely used to distinguish between cellular epidermis and ECM-rich dermis of mammalian skin. Here we used human skin biopsies to characterise: i) the ability of commonly used X-ray contrast agents (phosphotunstic acid [PTA] and inorganic iodine [Lugol’s solution; I_2_KI]) to enhance differentiation of these tissue regions and ii) the effects of X-ray tomography (of both unexposed and PTA- or I_2_KI-exposed biopsies) on subsequent H&E staining. Using both PTA and I_2_KI contrasting agents, good image contrast was achieved between the epidermis and dermis in scans of less than 4.5 hours duration (Fig. 7A). However, as previously reported^5^ tissue penetration of PTA was slow. Full penetration of a 3 mm diameter skin biopsy was not achieved after 40 hours exposure (Fig. 7B). Furthermore, whilst PTA exposure inhibited subsequent H&E staining, both epidermal and dermal cells and the dermal matrix were clearly stained following X-ray and I_2_KI + X-ray exposure. This inhibition of H&E staining may be due to blockage of aluminium binding sites (which are utilised by Harris’ Haematoxylin). The success of H&E staining post-I_2_KI exposure could be due to a lack of interaction between iodine and aluminimum binding sites or the loss of iodine ions prior to H&E staining.

**Figure 8:**
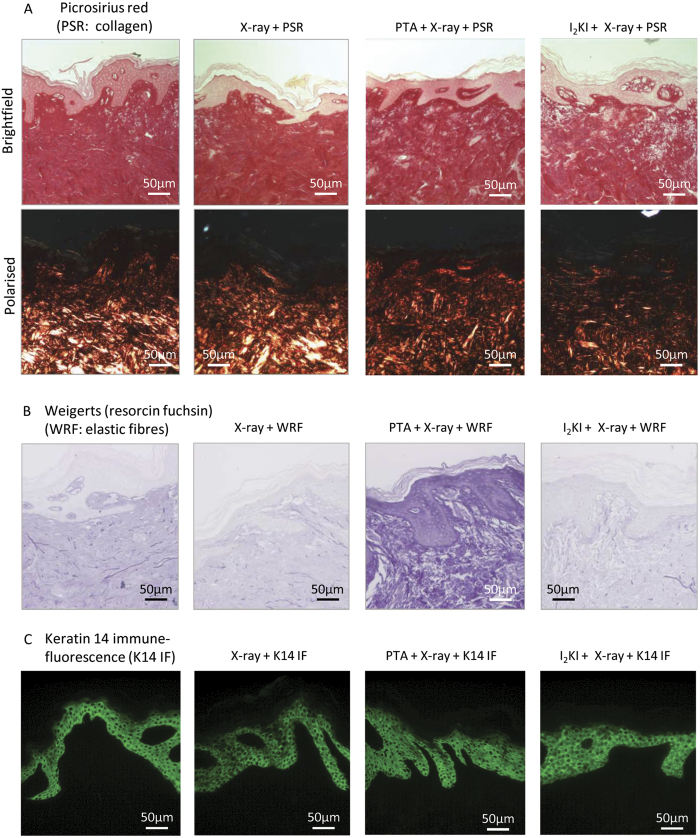
The effects of X-ray tomography on subsequent histology and immunofluorescence of structural components of the skin. Staining of tissue sections, from skin exposed to X-ray, for collagen show that prior staining with PTA or I_2_KI does not prevent the visualisation of collagen fibres (**A**). However, Weigert’s staining of skin for elastin fibres is disrupted by the presence of PTA (**B**). Unlike Weigert’s, exposure to X-ray and PTA or I_2_KI does not prevent the binding of Keratin 14 antibody to its epitope in skin, confirming that immunofluorescence is possible following X-ray tomography (**C**).

Whilst it is important to show that a widely used stain such as H&E is compatible with prior exposure to X-rays and X-ray contrast agents. This stain has low tissue specificity. The dermal ECM in human skin is composed primarily of fibrillar collagens and elastic fibres^48^. These supra-molecular assemblies can be specifically visualised by picrosirius red (PSR) staining and polarised light microscopy and by bright-field light microscopy of Weigert’s resorcin fuchin stained tissue respectively^49^. Fibrillar collagens were readily identified in sections cut from X-ray exposed biopsies and the binding of PSR to basic groups in the collagen triple helix^50^ appeared unaffected by prior exposure to PTA or I_2_KI (Fig. 8A). However, the staining of elastic fibres using Weigert’s (resorcin fuchin) stain which relies on the binding of iron, was again disrupted by the presence of PTA (Fig. 8B). It appears therefore that although X-ray exposure alone has little or no discernible effect on subsequent histological analysis the compatibility of specific combinations of X-ray contrast agent and histological stain may need to be established on a case-by-case basis. Crucially however, we also build on the observations of Chen and colleagues, who showed that microCT is compatible with immunohistochemical analyses in frog cartilage^51^, to demonstrate that an anti-keratin 14 antibody can successfully and specifically bind to epidermal epitopes in human skin following exposure to either X-rays alone or to both X-rays and X-ray contrast agents (Fig. 8C).

## Conclusions

Here we show that major tissue structures may be resolved by sub-micron X-ray tomography of native tissues and organs and that histological and immunohistochemical analysis of tissue structure and composition is compatible with prior exposure to X-rays and some X-ray contrast agents. In the specific case of the pressurised artery we demonstrate that static intra-luminal pressure has a differential effect on the morphology and hence volume of the medial and adventitial layers.

It is clear therefore that, with little user intervention, microCT and nanoCT in combination can rapidly visualise the 3D structure of relatively large tissue volumes (up to ~10 mm^3^) at sub-μm spatial resolutions. Compared with complementary 3D visualisation approaches, such as serial-sectioning combined with light or electron microscopy, or more recently developed technologies such as serial block face / SEM approaches, X-ray tomography not only maintains the same spatial resolution in all three axes but avoids artefacts induced by the loss, distortion and miss-alignment of sections^52^. In addition, as X-ray tomography is non-destructive, it can be used to identify volumes of interest for subsequent complementary microscopical and analytical analysis within a correlative tomography framework^53^. A key area for improving the technique would be to image native tissues. Although, in the current study, the use of chemical fixatives and a mechanical paraffin support provide a stable medium for the collection of high quality (without movement artefacts) 3D data, chemical fixation can induce both micro- and non-structural remodelling^54^,^55^. Previously microCT has been employed to characterise short-term (milliseconds) mechanical behaviours of metals and long-term (days) developmental remodelling in living insects^56^,^57^. It remains to be determined if microCT of native mammalian tissues is capable of resolving acute structural remodelling events in tissue sub-structure(s) as a consequence of dynamic physiological strains.

## Materials and Methods

### Tissue preparation, X-ray contrast agent exposure and histological and immunohistochemical staining

All procedures accorded to the UK Animals (Scientific Procedures) Act 1986 and were approved by the University of Manchester ethical review process. Pressure myography is conventionally used to study small arteries^58^,^59^,^60^. In this study we adapted the technique by using stronger sutures, larger cannulas and a surgical knotting technique to secure the rat CCA^61^. Left and right CCAs were dissected from 250-300 g male Wistar rats (Charles River, UK). The right CCA was mounted onto a pressure myograph (CH-1-QT, Living Systems: Vermont, USA). The left CCA was chemically fixed in the unpressurised state but the right CCA was subjected to an intra-luminal pressure of 110 mm Hg (equivalent to mean arterial pressure)^62^ which was maintained during fixation. All arteries (left and right CCA and descending thoracic aorta) were chemically fixed in 4% paraformaldehyde for two hours prior to ethanol dehydration and paraffin wax embedding using tygon tubing as a mould (Internal diameter 1.6 mm, Harvard Apparatus, Kent, UK)^63^,^64^. The dimensions of the tubing were chosen to minimise the thickness of the external paraffin wax. Finally wax cylinders were glued onto the head of metal pins so that the vessel axis could be mounted perpendicular to the X-ray source.

The 3D structure of human skin and the effects of X-ray exposure and X-ray contrast agents on subsequent histological and immunohistochemical staining procedures were characterised using buttock skin punch biopsies (obtained with informed consent and local ethical approval from the North West Research Ethics Committee [REC] reference 07/Q1409/9) and breast skin samples donated to the Manchester Skin Health Biobank (REC reference 09/H1010/10). All experiments were carried out in accordance with the approved local REC guidelines. Following fixation in Bouin’s solution, skin samples were either: i) stained with Lugol’s solution (I_2_KI) for 16 hours followed by washing in water; ii) stained with phosphotungstic acid (PTA) for 40 hours followed by washing in 70% ethanol, or iii) left unstained and submerged in 70% ethanol for 16 hours^5^.

### X-ray tomography and data processing

CCAs were imaged using a Carl Zeiss Xradia Versa-510 system (Carl Zeiss: California, USA) with the X-ray source voltage and current set to 60 kV and 83 mA, respectively. The detector and source were positioned ~35 mm and ~10 mm respectively from the sample to achieve a small amount of X-ray phase contrast using the inline method with the 4x objective^30^,^65^. Two successive scans were performed for each sample: i) a low resolution data collection scan of the complete artery cross-section using the 4x objective which achieved a voxel size of 0.75 μm and; ii) a higher resolution region-of-interest scan of a smaller tissue volume which employed the 20x objective and achieved a voxel size of 0.50 μm (detector to sample and sample to sources distances: 6.5 mm and 18 mm respectively). This improved resolution using the 20x objective (Fig. 2D) would be necessary to measure the dimensions of key structures such as the medial elastic lamellae. Exposure times per radiograph were in the range 6-10 s for the 4x scan, and 26-35 s for the 20x scan. Approximately 2000 projections were collected for each scan as the sample rotated over 360^o^. Further steps were taken to ensure good data quality; prior to scanning, each sample was placed within the scanner chamber overnight to ensure the sample and mount had attained thermal equilibrium (28 °C). Furthermore, the source was switched on for at least 30 minutes prior to each scan to improve stability.

Multiscale X-ray tomography imaging of the artery samples was carried out using Carl Zeiss Xradia Versa-510 (microCT) and Carl Zeiss Xradia Ultra-810 (nanoCT) systems. Gold beads of diameter 1-3 μm were placed on the sample to enable the same region to be identified from all scans. The aorta sample was scanned at two resolutions with the Versa XRM. The first, using the 4x objective, enabled the whole vessel cross-section to be imaged at 0.94 μm voxel size. A second, higher resolution region-of-interest, scan was carried out using the 20x objective yielding a voxel size of 0.5 μm. For both scans, the source voltage was set to 60 kV and 2001 projections were taken over 360° with and exposure time of 4 s and 30 s respectively. Nano-scale imaging of the elastic lamellae and the inter-lamellar regions was then carried out using the Ultra-810. This system is an X-ray microscope based on zone plate optics and has two magnification modes giving 150 nm and 50 nm resolution respectively. The system uses X-rays with energy 5.4 keV and can achieve Zernike phase contrast via the use of a phase ring^35^. A 65 × 65 × 65 μm region-of-interest of the sample was scanned at 150 nm resolution using Zernike phase contrast and 1851 projections were taken over 180° with an exposure time of 40 s/projection.

Buttock skin biopsies were imaged on a Carl Zeiss Xradia MicroXCT-400 system using the 4x objective, with a source voltage of 40 kV, a source current of 250 μA and an exposure time of 30 s/projection. The sample was positioned 8 mm from the source, and 45 mm from the detector. X-ray contrast-enhanced (I_2_KI or PTA) breast skin samples were imaged on a Carl Zeiss Xradia Versa-510 system also using the 4x magnification. I_2_KI stained tissue was imaged with 3201 projections at an exposure time of 1.5 s/projection, whilst PTA-stained tissue required only 601 projections at 1 s/projection (due to greater contrast). Following data acquisition, volumetric data was reconstructed from the scan data using the FDK algorithm^66^.

### Segmentation and morphological characterisation of 3D arterial reconstructions

Prior to segmentation, noise in the data was reduced by applying a 1D bilateral filter in the axial direction^67^. The vessel wall was then segmented in Avizo 8 using a conventional thresholding approach which relied on differences in X-ray density (Fig. 3). However, as the component medial and adventitial layers exhibited very similar X-ray densities, we developed a novel semi-automatic segmentation technique which exploited differences in grey-level texture in the two layers. Specifically, the elastic lamellae in the medial layer form concentric rings in the trans-axial plane, which become approximately straight lines when the vessel is digitally ‘unwrapped’; whereas the structures in the adventitial layer are less well ordered. This segmentation protocol was applied to each trans-axial slice in the 3D data set and comprised the following stages (as depicted in Fig. 3). First, the vessel wall was unwrapped by applying a smoothing spline (fitted to the image coordinates of the lumen edge) and resampling lines perpendicular to the spline curve at equal intervals. Next, the wall was segmented from the supporting paraffin background by applying a grey level threshold. The main adventitial features (radial connection and small groups of connected pixels) were then removed by applying a 1D morphological opening operation along the vertical axis (Fig. 3). Finally, the boundary between the medial and adventitial layers was established. The outer internal elastic lamina was located by finding, for each row, the outer most segmented pixel (with coordinate *xe*). In order to deal with residual adventitial features (which can cause deviations in the boundary position) an optimisation approach was adopted, based on the fast marching method^68^,^69^, which penalises large deviations from a vertical path. This method is related to Dijktra’s method for computing the shortest path in a network, and in this case was used to find the geodesic path through a weighted image. The weighted image was constructed by first determining the mean edge position along the horizontal axis, 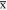 by fitting a Gaussian mixture model to the distribution of *xe* + 1. Pixels with coordinates *xe* + 1 that were close to 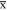 were given extra weight; whereas segmented pixels from the initial thresholded image were given a lower weight. A geodesic path (which is attracted to large weights and equates to the contour followed by the medial adventitial layer) can then be extracted. This contour can then be wrapped onto the original trans-axial slices by applying the inverse of the coordinate transform found in the first step. Finally, the surface of the medial layer boundary in the 3D stack was smoothed to remove noisy features. The complete process was implemented in Matlab 2013a (Mathworks, Massachuesetts, USA). Some manual adjustments to the segmentation were necessary in regions where the contrast was poorer, and this was achieved using the segmentation tools in Avizo 8. Despite this need for occasional manual intervention, in general, segmentation of wall layers within a single microCT data set can be achieved within 2-3  hours.

### Histological and immunohistochemical analysis

The effects of sectioning and intra-luminal pressure on the rat CCA was characterised by hematoxylin and eosin (H&E: Sigma Aldrich, Milton Keynes, UK) staining of 5 μm thick paraffin wax sections. Bright field optical images were captured using a Biozero-8000 fluorescence microscope (Keyence; Osaka, Japan). Subsequent to X-ray tomography (of both native and I_2_KI- or PTA- exposed), breast skin samples were sectioned, dewaxed, rehydrated and stained for cells and ECM (by H&E; Harris’ Haematoxylin, Eosin Y, Sigma Aldrich, Dorset, UK), fibrillar collagens (picrosirius red; 0.1% Sirius red F3BA (Sigma Aldrich) in saturated aqueous picric acid for 1 hour followed by a brief rinse in 0.1% acetic acid ) and elastic fibres (Weigert’s resorcin fuchsin; (Clin-Tech Ltd, Guilford, UK; 30 minutes in Weigerts and then rinsed in IMS, and then in water)^49^. Sections were dehydrated and mounted with DPX mounting media (Sigma-Aldrich) before imaging. Picrosirius red staining was visualised using polarised light microscopy (Leitz DMRB microscope: Leica, Milton Keynes, UK) with an Infinity X camera (DeltaPix: Maalov, Denmark), other stains were visualised using the All-in-one Type Fluorescence Microscope Biozero-8000 (Keyence; Osaka, Japan). For antigen retrieval and immunohistochemistical localisation of epidermal Keratin-14, paraffin sections were dewaxed, rehydrated, exposed to citrate buffer, permeabolised for 10 minutes at room temperature, washed in tris-buffered saline (TBS; 100 mmol l^−1^ Tris, 150 mmol l^−1^ NaCl; pH 7.4) and then exposed to a rabbit anti-keratin-14 primary antibody (1/1000; clone PRB-155P; Covance, New Jersey, USA) for an hour at room temperature. Sections were then washed in TBS before the addition of 488 AlexaFluor secondary goat anti-rabbit antibody (1:500; Invitrogen, Paisley, UK) for an hour. The sections were then washed and mounted with Fluoromount™ (Sigma-Aldrich).

## Additional Information

**How to cite this article**: Walton, L. A. *et al.* Morphological Characterisation of Unstained and Intact Tissue Micro-architecture by X-ray Computed Micro- and Nano-Tomography. *Sci. Rep.* **5**, 10074; doi: 10.1038/srep10074 (2015).

## Supplementary Material

Supplementary Information

Supplementary Video 1

Supplementary Video 2

Supplementary Video 3
