# Supplementary material for: Morphological Characterisation of Unstained and Intact Tissue Micro-architecture by X-ray Computed Micro- and Nano-Tomography
Source: Sci Rep. 2015 May 15;5:10074. doi: 10.1038/srep10074 (PMC4650804; doi:10.1038/srep10074)
Supplement: Supplementary Information [file srep10074-s1.pdf]

Morphological Characterisation of Unstained and Intact Tissue Micro-architecture by X-ray Computed Micro- and Nano-Tomography

Lucy A. Walton[1#], Robert S. Bradley[2#], Philip J. Withers[2], Victoria L. Newton[3], Rachel E.B. Watson[3], Clare Austin[1, 4§] and Michael J. Sherratt[3§*].

Supplementary Material

Video S1: 3D reconstruction of an unpressurised rat common carotid artery. The cylindrical artery tilts to reveal the inner luminal surface and internal wall structure. The integrity of the tissues and hence reconstruction is evident as virtual slices are sequentially removed. The reconstruction is then rebuilt from segmented adventitia (grey) and media (yellow). Finally, a portion of the artery wall is cut away, the vessel is rotated to reveal the adventitial surface before the camera zooms to magnify portion of the internal vessel wall.

Video S2: Cross-sectional virtual slices through an X-ray tomogram of human skin. Three hundred slices from a tomogram of human buttock skin. Cycling through the slices highlights undulations in both the X-ray dense outer *stratum corneum* and the dermal epidermal junction. Also evident are large hair follicles and obliquely oriented and X-ray dense keratin hair shafts. The dark filamentous features in the dermis may correspond to the dermal micro-vasculature.

Video S3: Virtual slices parallel to the skin surface. Computational sectioning of the same tomographic volume as depicted in Video S2, highlights the squamous appearance of the skin surface, the transition from the relatively X-ray translucent, cell-rich, epidermis to the X-ray opaque, ECM-rich, dermis and the remarkable X-ray density of the keratin hair shafts (white circles).
